# Economic Recession and Obesity-Related Internet Search Behavior in Taiwan: Analysis of Google Trends Data

**DOI:** 10.2196/publichealth.7314

**Published:** 2018-04-06

**Authors:** Ho-Wei Wang, Duan-Rung Chen

**Affiliations:** ^1^ Institute of Health Policy and Management National Taiwan University Taipei Taiwan; ^2^ Institute of Health Behaviors and Community Sciences National Taiwan University Taipei Taiwan

**Keywords:** obesity, economic recession, Google Trends, fast food, internet search, health-seeking behaviors, infodemiology

## Abstract

**Background:**

Obesity is highly correlated with the development of chronic diseases and has become a critical public health issue that must be countered by aggressive action. This study determined whether data from Google Trends could provide insight into trends in obesity-related search behaviors in Taiwan.

**Objective:**

Using Google Trends, we examined how changes in economic conditions—using business cycle indicators as a proxy—were associated with people’s internet search behaviors related to obesity awareness, health behaviors, and fast food restaurants.

**Methods:**

Monthly business cycle indicators were obtained from the Taiwan National Development Council. Weekly Taiwan Stock Exchange (TWSE) weighted index data were accessed and downloaded from Yahoo Finance. The weekly relative search volumes (RSV) of obesity-related terms were downloaded from Google Trends. RSVs of obesity-related terms and the TWSE from January 2007 to December 2011 (60 months) were analyzed using correlation analysis.

**Results:**

During an economic recession, the RSV of obesity awareness and health behaviors declined (*r*=.441, *P*<.001; *r*=.593, *P*<.001, respectively); however, the RSV for fast food restaurants increased (*r*=−.437, *P*<.001). Findings indicated that when the economy was faltering, people tended to be less likely to search for information related to health behaviors and obesity awareness; moreover, they were more likely to search for fast food restaurants.

**Conclusions:**

Macroeconomic conditions can have an impact on people’s health-related internet searches.

## Introduction

### Background

Obesity is highly correlated with the development of several chronic diseases, including cardiovascular disease and type 2 diabetes [1,2]. According to the World Obesity Federation, if levels of obesity and overweight status continue to rise, 2.7 billion adults will be overweight by 2025, up from 2 billion in 2014 [3]. In Taiwan, the prevalence of individuals defined as overweight and obese is also increasing with an estimated prevalence of 48.9% in males and 38.3% in females [4]. Taking the obesity rates of both adults and children into account, Taiwan ranks highest in Asia, suggesting a critical public health issue that must be solved decisively [4].

### Previous Research

Studies have found that the prevalence of obesity shifts toward lower socioeconomic status groups as a country’s gross national product increases [5,6]. Studies conducted in the United States and Europe have determined that the highest obesity rates are associated with the lowest income levels [7,8]. One reason for increased obesity among those with lower incomes is that a lower income is often associated with poor health behavior and dietary quality [9,10].

Several studies have also indicated a potential association between macroeconomic conditions and obesity-related health behavior. For example, one study used a convenience sample from a metropolitan city in the US Midwest and discovered that an economic downturn may increase rates of obesity by forcing families to cut their food expenditure [11,12]. Moreover, the downturn motivated consumers to switch to lower-priced and higher-calorie foods, which usually have higher fat and sugar content while containing lower quantities of recommended micronutrients [11,12]. Another study in the United States found that recession-driven unemployment was associated with an increase in higher-calorie purchases [13]. Other studies, using the US Behavioral Risk Factor Surveillance System data, revealed that a recession can lead to a lower dietary quality and less physical activity [14,15]. However, other studies have indicated that people have more time to exercise and prepare healthy meals during economic downturns and are thus more likely to maintain a healthy weight [16,17].

The most recent global recession (Great Recession, 2008-2009) was the most severe, as measured by duration, since the Great Depression of the 1930s [18]. In Taiwan, several indices reached record lows during the most recent recession. Car sales declined 28.09% in September 2008, retail sales fell to a 7-year low in September 2008 (–5.06% year-on-year growth), the unemployment rate soared to a 4-year high (4.27%), and the overall consumer confidence index dropped to 79.56 [19,20]. All of these figures revealed that consumer spending had been retrenched by the decline in income [19,20]. Although various sources of conflicting evidence regarding the effect of the recession on health behaviors remain controversial, recessions still provide a unique opportunity to shed light on the association between economic conditions and the risk of obesity.

Individuals today often use the internet and various search engines, such as Google and Yahoo, to obtain information to support their decisions [21]. These online searches through search engines create trend data, which can be analyzed in real time [22,23]. Since 2004, Google has provided two services for trend analyses: Google Flu Trends and Google Trends. Google Flu Trends has been identified as a powerful tool used in influenza surveillance in the United States, identifying influenza epidemics up to 7-10 days before detection by the Centers for Disease Control and Prevention’s influenza surveillance network [22]. During periods of infectious disease prevalence, Google Flu Trends has also been able to predict the volume of emergency department visits [24,25]. However, Google Flu Trends failed to correctly estimate the scale of the 2009 H1N1 pandemic in the United States. Several mistakes that led to Google Flu Trends’ overestimation of H1N1 incidence were caused by the limited transparency of Google’s treatment of data and its dynamic algorithm, which was due to Google’s business considerations [26,27]. The Google Flu Trends service is no longer available in many countries.

Google Trends also has predictive capacity for monitoring the epidemic curves of food-borne illnesses, such as peanut butter-associated outbreaks of *Salmonella enterica* serotype Typhimurium [28] and the incidence of human immunodeficiency virus [29,30]. With regard to population health concerns during the Great Recession, a study using Google Trends found that specific searches with the greatest relative excess were stomach ulcer and headache symptoms [31].

### Research Goals

The purpose of this study was not to contest or generate additional theories to support the contentious link between macroeconomic conditions and obesity-related health behaviors, but rather it was to understand how an economic downturn might affect obesity-related internet search behavior. Thus, by using Google Trends we investigated how changes in economic conditions—using business cycle indicators as a proxy—affected people’s internet search behaviors, including searches related to obesity awareness, obesity-related health behavior, and fast food restaurants.

## Methods

### Data Sources

#### Great Recession Period

**Figure 1:**
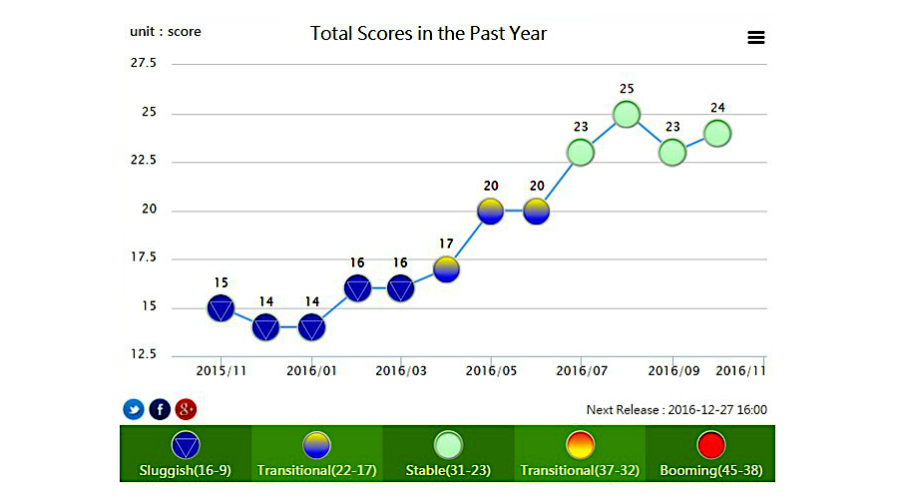
Taiwan business cycle indicators.

The 2008-2009 economic recession was the most severe since the Great Depression of the 1930s; therefore, it provides an excellent opportunity to study how the business cycle affects internet search behaviors for health-related issues [18]. Business cycle indicators, released monthly by the Taiwan National Development Council, are used to measure economic development [32]. The scores of indicators represent five different levels of economic prosperity, including sluggish (scores of 9-16), downward transition (scores of 17-22), stable (scores of 23-31), upward transition (scores of 32-37), and booming (scores of 38-45) [32] (see Figure 1 for further details).

**Figure 2:**
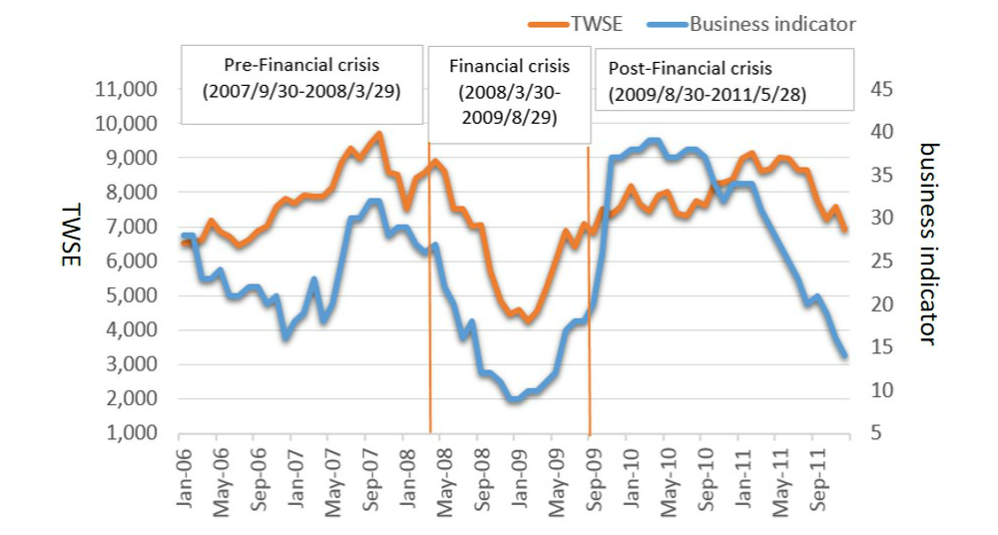
Time series for the monthly business cycle indicator and Taiwan Stock Exchange Weighted Index (TWSE) between January 2006 and December 2011.

In this study, the recession period was defined as the time when the business cycle indicator dropped below the stable stage, climbed to the transitional stage (above 23) from April 2008 through September 2009, and then returned to growth until December 2011 (see Figure 2 for graphical data).

#### Google Trends Search Terms and Trends

Google Trends analyzes all search queries of a specific term to quantify interest in topics at the population level, thus serving as an increasingly useful research tool. Relative search volume (RSV) is the proportion of searches for a given term out of all searches for a given geographic location and time period, which is then normalized to a 0-100 scale [33,34]. To capture a broad sense of the search terms regarding obesity-related conditions and behaviors, 49 search terms were cataloged through Google Trends; the relevant terms were derived from Google Trends’ explore function [31]. The health belief model (HBM) is a psychological health-behavior-change model developed to explain and predict health-related behaviors, in particular the uptake of health services [35]. HBM has been applied to understand patients’ responses to symptoms of disease, compliance with medical regimens, and lifestyle behaviors. In this study, we classified these key search terms into three categories: (1) obesity-related awareness terms, such as metabolic syndrome; (2) obesity-related health behavior terms, such as healthy diet; and (3) fast food restaurant terms such as McDonald’s (Table 1).

Google Trends only reports results above a certain threshold. When Google Trends cannot report the search volume for a term, the message displayed is, “Not enough search volume to show graphs.” Thirty-four search terms had insufficient search volume. Weekly data on the remaining 15 search terms from January 2007 to December 2011 (60 months) were accessed and downloaded from Google Trends on June 28, 2016. Table 1 displays the list of obesity-related search terms categorized into three groups. Multimedia Appendix 1 lists these terms in Chinese, along with their English equivalents.

**Table 1:** List of obesity-related search terms.

Category	Search terms
Awareness terms	Obesity; metabolic syndrome; endocrinology; triglycerides; body fat percentage; body mass index
Health behavior terms	Healthy diet; aerobics; effective diet; meal replacement; on a diet; fitness
Fast food restaurant terms	McDonald’s; KFC; pizza

#### Taiwan Stock Exchange Weighted Index

As stock market volatility is a predictor for macroeconomic volatility [36,37], we demonstrated the relationship between the Taiwan Stock Exchange (TWSE) and the RSVs of obesity-related terms during three business cycles. Weekly TWSE data were accessed and downloaded from the Taiwan Stock Exchange Corporation on June 30, 2016 [38].

### Statistical Analyses

Pearson correlation coefficient analysis is widely used in Google Trends research [39]. To assess the strength of the linear relationship between the RSVs and TWSE, a Pearson correlation coefficient analysis with 95% CI was reported. The correlation between the RSVs and TWSE was analyzed first and then followed by lead pattern analysis. The TWSE’s lead pattern analysis was then analyzed by 1-month and 3-month intervals. For example, a 1-month lead evaluated the correlation of the TWSE from January 2009 with obesity-related searches in February 2009. All statistical analyses were conducted using IBM SPSS Statistics for Windows, version 20.0 (IBM Corp, Armonk, NY); a two-tailed *P* value of less than .05 was required for statistical significance in all analyses conducted.

## Results

**Figure 3:**
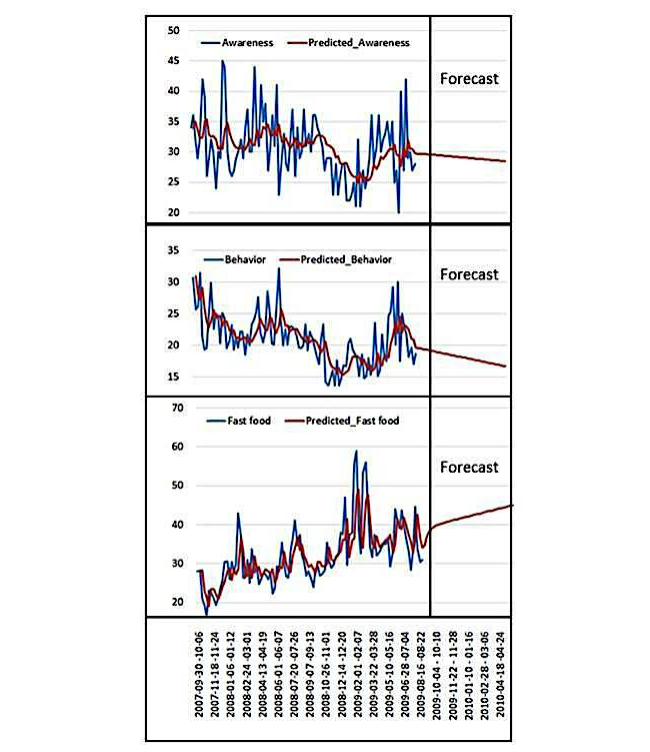
Time trends for queries of the three categories around the Great Recession.

Table 2 lists the change of RSVs by categories. The RSV of obesity awareness terms showed a continual downward trend across the three periods. Despite the upturn in the financial market, the RSV of obesity awareness terms became weaker during the postfinancial crisis period, and the RSV of health behavior terms fell dramatically (by 12%) during the financial crisis period. However, the RSV of fast food restaurants increased sharply during this period, rising by 27% (see Figure 3). Autoregressive integrated moving average (ARIMA) models were applied to predict the tendency by the end of April 2010. The results reported that awareness and behavior terms trended down, while fast food terms were showed an upward trend (see Figure 3).

Figure 3 shows time trends before and during the Great Recession for all three categories of search terms that were analyzed. Each line indicates a specific query trend, with the red line indicating the tendency across all queries based on ARIMA models.

### Nowcasting Effect

Nowcasting, also known as contemporaneous forecasting, is used to describe the extent of a current association. During the prefinancial crisis period, the RSV of health behaviors was positively associated with the TWSE (*r*=.504, *P*=.009), suggesting that health behavior searches increased while the TWSE went up. The RSV of fast food restaurants was negatively associated with the TWSE (*r*=−.529, *P*=.006), indicating that fast food restaurant searches increased while the TWSE declined.

Second, during the period of the financial crisis, all RSVs of the three categories were correlated with the TWSE. The RSV of obesity awareness and health behavior declined while the TWSE declined (*r*=.441, *P*<.001; *r*=.593, *P*<.001, respectively), and fast food restaurant searches increased while the TWSE declined (*r*=−.437, *P*<.001).

Third, the RSV of obesity awareness and health behavior correlated with the TWSE after the financial crisis, but the correlation coefficient changed from a positive to a negative direction (*r*=−.544, *P*<.001; *r*=−.548, *P*<.001). This finding may suggest that people were less concerned with obesity-related issues when job opportunities returned during the postfinancial crisis period (see Table 3).

### Forecasting Effect

In addition to the nowcasting effect, the forecasting effect can be used to determine the TWSE’s lead pattern over the search trends. As aforementioned, for example, a 1-month lead TWSE represented the correlation between the TWSE in January 2009 and obesity-related searches in February 2009. All figures are reported in Table 3.

**Table 2:** Relative search volumes (RSVs) by categories.

Time period	Search volume indexes (RSVs)		
	Awareness (% change)	Behavior (% change)	Fast food (% change)
Reference (before prefinancial crisis; 2006/10/1-2007/9/29)	34	23	23
Prefinancial crisis (2007/9/30-2008/3/29)	32 (–6)	23 (0)	27 (16)
Financial crisis (2008/3/30-2009/8/29)	30 (–6)	20 (–12)	34 (27)
Postfinancial crisis (2009/8/30-2011/5/28)	29 (–3)	19 (–5)	38 (12)

**Table 3:** Pearson cross-correlation analysis of search terms and Taiwan Stock Exchange (TWSE), 2007-2011.

Attribute and time period		TWSE preceded search terms by time (*P* value)		
		No lag	1 month	3 months
**Obesity awareness**				
	Prefinancial crisis (2007/9/30-2008/3/29)	.277 (.170)	.086 (.677)	.087 (.674)
	Financial crisis (2008/3/30-2009/8/29)	.441 (<.001)	.453 (<.001)	.318 (.006)
	Postfinancial crisis (2009/8/30-2011/5/28)	­–.554 (<.001)	–.595 (<.001)	–.354 (.001)
**Health Behaviors**				
	Prefinancial crisis (2007/9/30-2008/3/29)	.504 (.009)	.421 (.032)	.344 (.086)
	Financial crisis (2008/3/30-2009/8/29)	.593 (<.001)	.646 (<.001)	.388 (.001)
	Postfinancial crisis (2009/8/30-2011/5/28)	–.548 (<.001)	–.405 (<.001)	–.298 (.004)
**Fast Food**				
	Prefinancial crisis (2007/9/30-2008/3/29)	–.529 (.006)	–.640 (<.001)	–.028 (.893)
	Financial crisis (2008/3/30-2009/8/29)	–.437 (<.001)	–.513 (<.001)	–.530 (<.001)
	Postfinancial crisis (2009/8/30-2011/5/28)	.049 (.646)	.095 (.372)	.181 (.086)

First, during the prefinancial crisis period, a 1-month lead TWSE was statistically associated with the RSVs of health behaviors and fast food (*r*=.421, *P*=.032; *r*=−.640, *P*<.001, respectively). Second, during the financial crisis period, a 3-month lead TWSE was found to be significantly associated with the RSVs of all three categories (obesity awareness: *r*=.318, *P*=.006; health behaviors: *r*=.388, *P*=.001; fast food: *r*=−.530, *P*<.001).

For searches using obesity awareness and health behavior terms, the TWSE showed a 3-month predictive power with positive association, suggesting that the stock market collapse was followed 3 months later by a decline in interest for obesity awareness and health behaviors. These results imply that people were more likely to disregard obesity-related health issues when the business cycle fell into recession. Furthermore, the TWSE was negatively related to the RSV of fast food restaurants (*r*=−.530, *P*<.001), suggesting that searches for fast food were possibly caused by a sluggish economy and consequently limited budgets for healthy food.

Third, after the financial crisis period, although a business cycle and stock market turnaround took place, searches for obesity awareness and health behavior did not follow the same trend. In contrast, both categories of search terms demonstrated a negative correlation with TWSE, suggesting that obesity-related health issues may have been lower than before the economic downturn due to the economic recovery, which lead to job opportunity increases (lower unemployment).

**Figure 4:**
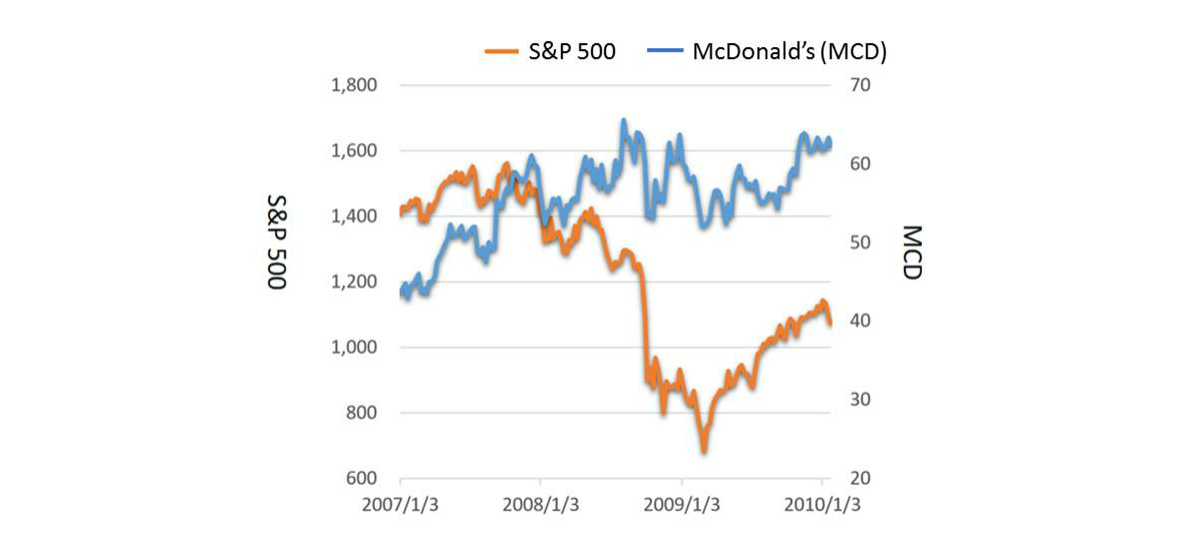
S&P 500 and McDonald’s Corp. (MCD:NYSE) share performance between 2007 and 2010.

Finally, fast food consumption has been assumed to be a potential risk factor for being overweight and obese, due to the high energy densities and high glycemic loads associated with such foods [40]. Research has suggested that an economic downturn pushes individuals to replace nutritious but expensive foods with cheaper and higher-calorie substitutes [15,41]. Such findings can partially be explained by lower budgets for food expenditure [11,42]. Moreover, commercial promotions by fast food companies during the recession periods might have triggered the consumption of fast foods; a trend that was indicated by the stock price of fast food companies (eg, McDonald’s) that bucked the negative trend while the major index continued to struggle (see Figure 4) [43,44].

Our findings support this relationship by demonstrating the negative correlation between TWSE and fast food searches during the financial crisis period (*r*=−.437, *P*<.001).

**Figure 5:**
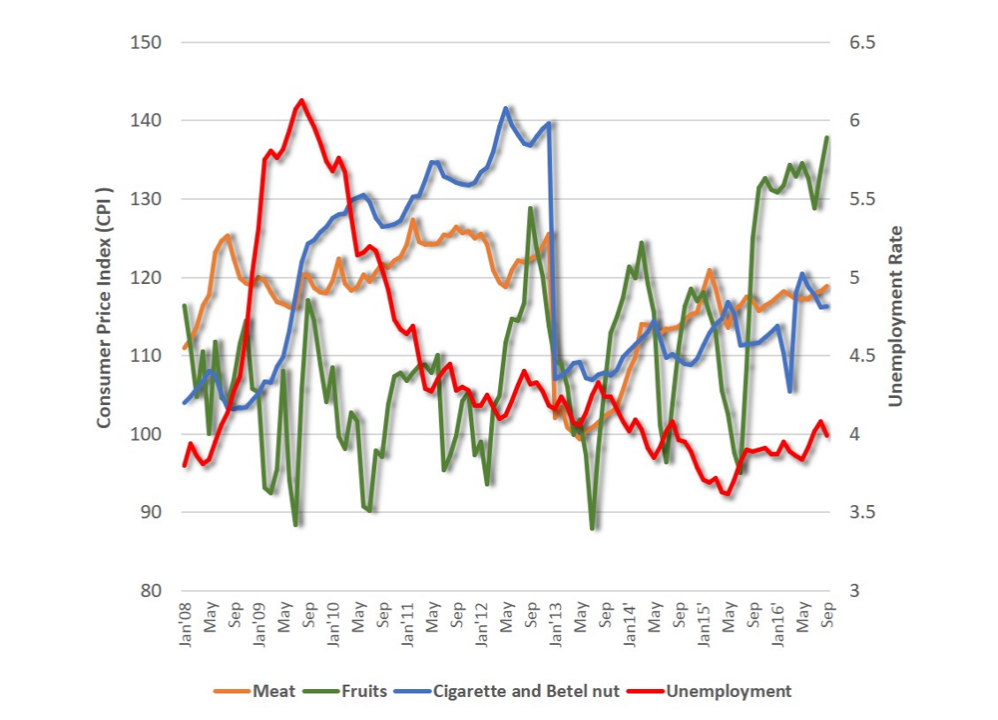
Consumer price index and unemployment trends, 2008-2016.

The consumer price index (CPI) tracks monthly data on changes in the prices paid by consumers for a specific or representative basket of goods and services [45]. We analyzed the relationship between unemployment and CPI for meat and fruit in Taiwan from 2008 through 2016 (see Figure 5); the results revealed a positive association between unemployment rate and meat consumption (*r*=.209, *P*=.032) and negative association between unemployment rate and fruit consumption (*r*=−.421, *P*<.001; see Table 4). Such a result echoes studies that indicated people increase caloric purchases, such as meat, for protein satiety during an economic downturn (high unemployment rate) and spend less on healthy foods, such as fruit [11,13,41,42]. Furthermore, the CPI for cigarettes and betel nuts (a commonly chewed nut in Taiwan with a mildly stimulating effect) also had a positive relationship with unemployment (*r*=.220, *P*=.024), which suggests unhealthy behavior may also rise when the economy and job market are sluggish.

**Table 4:** Pearson cross-correlation analysis of the correlation between consumer price index and unemployment for meat, fruit, and cigarettes and betel nuts (2008-2016).

Variable	Unemployment	Meat	Fruit	Cigarette and betel nuts
Unemployment	1.00			
Meat (*P* value)	.209 (0.32)	1.00		
Fruit (*P* value)	­–.421 (<.001)	.003 (.974)	1.00	
Cigarettes and betel nuts (*P* value)	.220 (.024)	.599 (<.001)	–.094 (.339)	1.00

## Discussion

### Principal Findings

This study contributes to an understanding of how business cycles are related to online searches for obesity-related awareness, health behavior, and fast food restaurants. The results revealed a positive association between the TWSE, obesity-related awareness, and health behavior searches during the prefinancial and financial crisis periods, suggesting that economic hardship may be the underlying force affecting individual obesity-related internet searches. Previous studies have indicated that individuals may engage in less physical activity or cease membership of health and sports clubs during periods of economic downturn [14,15,46]. This study revealed that during the postfinancial crisis period, the associations between the TWSE and searches for obesity awareness and obesity-related health behaviors changed from positive to negative, suggesting that as the TWSE increased, internet searches for obesity awareness and obesity-related health behaviors decreased. Such a finding might be explained by job stress or having less time for self-care activities during economic upturns [17,47]. However, despite the upturn in the financial market, the RSV of obesity awareness terms became weaker during the postfinancial crisis period (see Table 2). This finding may suggest that during the postfinancial crisis period, people had more job opportunities and less time to be concerned with obesity-related issues.

The results suggested that the performance of the TWSE moderately correlated with these search trends, demonstrating the possibility that monitoring the TWSE could plausibly predict future obesity-related searches on the internet. Additionally, the results also suggested that the TWSE demonstrated a 3-month leading effect; however, the 1-month leading effect was much stronger during the prefinancial crisis and financial crisis periods.

Finally, our results suggested that the TWSE could also play a role in monitoring changes in obesity-related aggregated individual search behaviors at population scale. If so, one of the primary indicators of economic prosperity not only represents the nation’s economy but is also a leading indicator of population health. This study provides a new methodological lens for improving the monitoring of obesity. This method can help governments recognize that recessions may result in an increase in obesity-related problems. While addressing the issues of a stalling job market and high unemployment, governments should also look to provide resources for dealing with obesity.

### Limitations

Due to the Google Trends algorithm, several limitations of this study should be noted. First, there was difficulty in identifying search trends that were generated by true cases. In particular, Google Trends tends to be influenced by media exposure of specific diseases (eg, drug advertisements), which drives more nonrelated individuals to search for terms and thus increases the search volume [48]. This limitation can possibly be more effectively controlled in future research [49]. Another limitation was that the calculation of Google Trends depends on Google’s assumptions and normalization, which are not clearly reported. Third, the method by which relevant terms are derived from Google Trends’ explore function has also not been disclosed by Google. Finally, potential alternative variables, such as increased advertising and low cost special offers by fast food companies during economic recessions may be confounders that influenced the results; this issue is worthy of study in future research.

### Conclusion

In conclusion, internet search data can be a potentially useful tool for health policy makers to identify obesity-related issues and possible obesity problems within the population. Google Trends serves as an easily accessible and real-time surveillance tool. Despite its limitations, this study highlighted Google Trends as a useful tool for establishing a plausible relationship between obesity-related search terms and macroeconomic events.

## Appendix

Multimedia Appendix 1 List of Chinese obesity-related search terms and their English equivalents.

## Abbreviations

ARIMA: autoregressive integrated moving average
CPI: consumer price index
HBM: health belief model
RSV: relative search volumes
TWSE: Taiwan Stock Exchange
