# Supplementary figures and images for: Economic Recession and Obesity-Related Internet Search Behavior in Taiwan: Analysis of Google Trends Data
Source: JMIR Public Health Surveill. 2018 Apr 6;4(2):e37. doi: 10.2196/publichealth.7314 (PMC5910536; doi:10.2196/publichealth.7314)

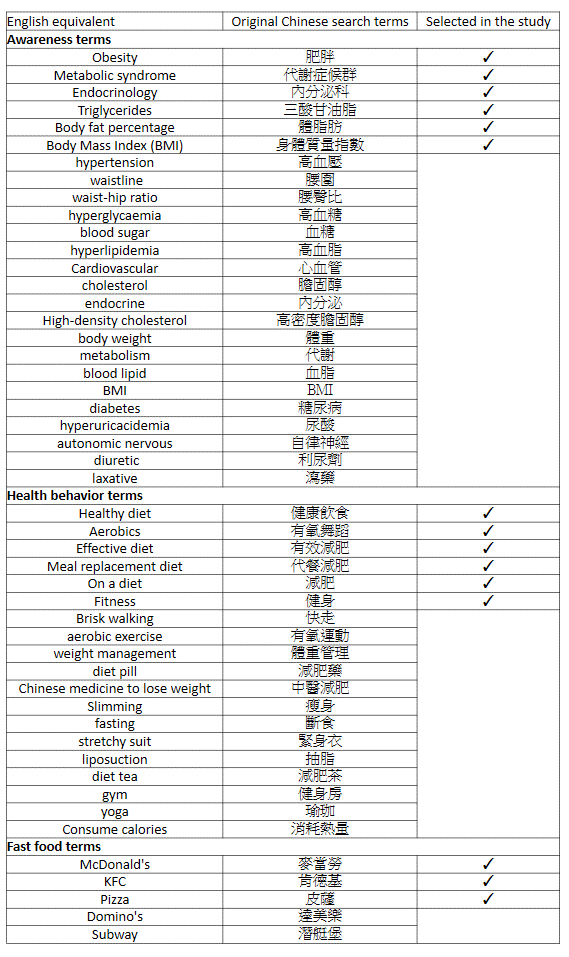

Supplement: Multimedia Appendix 1 [file publichealth_v4i2e37_app1.png]
